# The Pressing Issue of Micro- and Nanoplastic Contamination: Profiling the Reproductive Alterations Mediated by Oxidative Stress

**DOI:** 10.3390/antiox11020193

**Published:** 2022-01-19

**Authors:** Maria Carmela Ferrante, Anna Monnolo, Filomena Del Piano, Giuseppina Mattace Raso, Rosaria Meli

**Affiliations:** 1 Department of Veterinary Medicine and Animal Productions, University of Naples Federico II, Via F. Delpino 1, 80137 Naples, Italy; filomena.delpiano@unina.it; 2 Department of Pharmacy, University of Naples Federico II, Via D. Montesano 49, 80131 Naples, Italy; mattace@unina.it (G.M.R.); meli@unina.it (R.M.)

**Keywords:** microplastics, nanoplastics, reproduction, oxidative damage, aquatic organisms, terrestrial organisms, antioxidant substances

## Abstract

Micro- and nanoplastics (MPs/NPs) are among the most widely distributed pollutants in the environment. It has been suggested that exposure to MPs/NPs can trigger toxicity pathways among which inflammation and oxidative stress (OS) play a pivotal role. Once absorbed, MPs/NPs may act locally or access the bloodstream and, following the translocation process, reach several organs and tissues, including the gonads. Notably, MPs/NPs can bioaccumulate in human and murine placenta, opening new scenarios for toxicological evaluations. We review recent studies on the effects of MPs/NPs on the reproductive health in aquatic and terrestrial organisms of both sexes, focusing on the role of OS and the antioxidant defence system failure as the main underlying mechanisms. Alterations in gametogenesis, embryonic and offspring development, and survival have been shown in most studies and often related to a broken redox balance. All these detrimental effects are inversely related to particle size, whereas they are closely linked to shape, plastic polymer type, superficial functionalization, concentration, and time of exposure. To date, the studies provide insights into the health impacts, but no conclusions can be drawn for reproduction toxicity. The main implication of the few studies on antioxidant substances reveals their potential role in mitigating MP-induced toxic effects.

## 1. Growing Concerns on Effects of Micro/Nanoplastics Toxicity

During the last twenty years, increasing attention has been paid to plastic environmental pollution, which affects terrestrial and aquatic ecosystems and air [1], representing a threat to living organisms. This pollution is expected to grow in the near future [2].

Several studies have proposed classifications of plastics depending on their size. Even if there is not yet a general consensus, many researchers agree to define microplastics (MPs) as those in the range 100 nm–5 mm in diameter and nanoplastics (NPs) as those in the range 1–100 nm [3]. Based on their shape, they are mainly listed as pellets, fibres, fragments, spheroids, and granules. MPs largely include the following three polymer types: polyethylene (PE), polypropylene (PP), and polystyrene (PS) [3]. MPs may be divided into primary and secondary ones based on their origin [4]. Primary MPs are small, persistent, chemically inert, raw particles employed in several applications (i.e., personal care products, drug vectors, and synthetic fabrics). Deriving from anthropic activities, they are released directly into the environment through wastewater, sewage systems, and industrial discharge [5,6]. Secondary MPs originate from the fragmentation over time of plastic debris due to biodegradation processes, abrasion by the wind, or UV rays [4]. Both primary and secondary MPs tend to fragment in NPs. MPs/NPs toxicity is closely related to their bioaccumulation and size [7,8]. Several studies reported that entanglement by [9] and ingestion of anthropogenically induced plastic debris, or their capturing in gills [10], are the primary causes of MP damage in aquatic species. The trophic transfer among trophic levels, from producers to top predators [11], can lead to the biomagnification of MPs/NPs toxicity. Humans, as with other terrestrial mammals, are exposed to MPs/NPs primarily by ingestion of contaminated food (i.e., sea salt, sea products, beer, honey, and potable water) [12,13,14,15,16]. In the last years, chronic inhalation has been considered mainly for industrial and agricultural workers exposed to high concentrations of MPs/NPs (see references in [17,18]). Human exposure through indoor air also has been reported [19,20]. Moreover, NPs could cross the dermal barrier in mammals [21], even though this route of contact may be less significant than the oral or inhalation one. Besides the skin and mouth, hair has also proved to be a significant passive receptor [22].

MPs in the size range 0.1–150 µm, once absorbed, act locally or translocate from the gut to lymphatic and blood circulatory systems, reaching mammalian organs and tissues far from the site of exposure (see [3]). Therefore MPs/NPs may bioaccumulate in several organs, including female and male gonads [23,24,25].

MPs/NPs toxicity is influenced by plastic type, particle size, superficial functionalization, environmental conditions, species, experimental model used, time of exposure, route of exposure, and concentration/dose difference [7,17,21,26,27]. MPs can be fragmented and/or biotransformed into smaller particles (nanometre size); these modifications may occur in both abiotic matrices and living beings. In addition to size, surface chemistry and charge, the cellular uptake of NPs can modulate their interaction with biological molecules (i.e., phospholipids, carbohydrates, and proteins), modifying the NPs’ behaviour [28,29].

MPs/NPs toxicity is due to oxidative stress (OS), inflammation, cytotoxicity, immunotoxicity, and metabolic disorders, among the others [21]. Most studies indicate OS as the major mechanism responsible for MP/NP-induced damage. However, all the mechanisms are strictly interconnected with each other and the induction of one pathway can trigger or sustain the others.

The main MPs/NPs cytotoxic mechanisms are membrane injury and OS, and other ones are genotoxicity and immune response induction [30]. NPs uptake leads to a loss of cell membrane integrity, consequent pore formation, and increased intracellular reactive oxygen species (ROS) production from the mitochondria [31]. Increased ROS generation is in turn responsible of mitochondrial dysfunction and release of pro-apoptotic factors and pro-inflammatory cytokines, among others, which result in cell death [26]. Moreover, NPs impair membrane structure and function, modifying the lipid bilayer with an increased thickness and altered molecules flow [32].

MPs cytotoxic damage is also related to an increased expression of pro-inflammatory genes potentially responsible for pulmonary, dermal, and immune damages [33,34,35]. Inflammation induced by MPs causes an alteration in gut microbiota composition, increasing the serum cytokine levels, accompanied by the impairment of the innate and adaptive immunity [36]. The dysfunction of the immune response induced by NPs is related to the induction of the apoptosis process triggered by ROS production, the reduced phagocytosis, the increased lysozyme activity, and the modulation of transcription of genes implicated in the OS, inflammatory, and apoptotic pathways (i.e., *NF-kB*, *Bcl-2* and *Hsp90*) [37,38,39]. Indeed, several lines of evidence show a strict relationship between MPs/NPs toxicity and OS, identifying alterations in redox balance and an impaired response of the antioxidant defence system at the onset of the immune and nervous system damage, as well as carcinogenicity (see, for instance, [40]).

The detrimental effect of MPs/NPs on several organs and functions, including the reproductive ones, can be aggravated by the co-exposure to other toxic chemical xenobiotics adsorbed onto their surface. In fact, MPs/NPs can act as a vector for other pollutants, allowing their biomagnification (see [41] and references therein). In particular, the mix of MPs/NPs with additives (bisphenols or phthalates) and/or persistent organic pollutants (POPs) produces a higher toxicity than that due to MPs/NPs alone [42,43]. Pollutants adsorption may be increased by biofilms that are microorganisms colonizing MPs [44].

Co-exposure of clams to MPs and antibiotics (oxytetracycline and florfenicol) resulted in increased bioaccumulation of these drugs, revealing an enhanced toxicological potential and antibiotic resistance risk for human consumption of fishery products [45].

Despite the absence of evidence in humans, animal studies (particularly on marine species) indicate that MPs/NPs affect several reproductive parameters and functions in living beings. Very recently, preliminary evaluations of the effects of MPs exposure on mammalian reproduction have revealed the alterations in spermatogenesis/sperm quality and ovary in exposed animals and the indirect damages on the embryo and offspring occurring via gestational exposure [23,46,47].

Here, we review the recent literature data to better define the effects of MPs/NPs on reproduction in aquatic and terrestrial organisms, focusing on the role of OS as the underlying cross-sectional mechanism of toxicity. Studies regarding the co-exposure of chemical contaminants and MPs/NPs were also considered. Antioxidant substances with a role in the reduction of MP-induced damages were examined since such substances are molecules that hinder OS, preventing ROS overproduction.

In this review, we analysed the journal articles reported on PUBMED^®^ that comprises citations for biomedical literature from the MEDLINE database, life science journals, and online books. Moreover, we considered papers reported on Google Scholar, Web of Science, and Scopus (until October 2021), using as keywords: microplastics or nanoplastics, reproduction, oxidative stress, and antioxidant substances. We tried to identify the knowledge gaps in this new research field and provide recommendations to optimize further investigation.

## 2. Oxidative Stress and Inflammation in Micro/Nanoplastics Toxicity

MP/NPs-induced toxicity has been mainly investigated in aquatic organisms and only few experimental studies have been carried out on terrestrial species. All these studies deal with different biological structures—from cells (i.e., head-kidney leucocytes and fibroblasts [48,49] to tissues (e.g., [50,51]) and to the whole organism (e.g., [52,53])—through the evaluation of biochemical parameters [54]. However, most data are focused on the effects of PS particles, overlooking those of the other plastic types (i.e., PE and PP).

Recently, MP toxicity mechanisms were reviewed in terms of ecotoxicity and human health risk assessment [55]. It was suggested that the key event was ROS (i.e., hydrogen peroxide, singlet oxygen, superoxide anion, ozone, hydroxyl radicals, and nitric oxide) overproduction. ROS cause damage to cell components, including lipids, proteins, and DNA, and the adverse outcomes are a decrease in growth rates, reproduction failure, and increased mortality. Antioxidant enzymes counteract reactive species overproduction; however, when it overcomes the antioxidant power, oxidative damage may occur [56]. MPs/NPs can differently impact the antioxidant system. It is commonly assumed that smaller MPs are associated with elevated OS [57,58,59], even if contrasting results are also reported in the literature [60,61]. This variability can be attributed to (i) the type and size of the MPs used in the different experimental models; (ii) the organs, tissues or cell types examined; and (iii) the concentrations/doses used, which are typically higher than the environmentally relevant ones. Probably, low MPs/NPs contamination could not be enough to trigger a response, but its extent is expected to increase in the near future [2].

Recently, the effects of short- and long-term exposure (3 and 13 days) to PS-MPs/NPs of different sizes (65 nm, 100 nm and 1µm) and concentrations (1 and 10 mg L^−1^) on growth and OS-related parameters were assessed for the dinoflagellate *Karenia mikimotoi* [62]. PS-NPs induced a major growth inhibition, probably caused by physical blockage and membrane damage due to the aggregation of NPs to microalgae. The smaller particles crossed the membranes through pores allowing the leakage of cytoplasm, generating a greater cytotoxic effect. Moreover, PS-NPs caused the most severe effects on catalase (CAT) and superoxide dismutase (SOD) activity and malondialdehyde (MDA) content and ROS production.

A significant augmentation of lipid peroxidation (LPO), estimated as thiobarbituric acid reactive substances, was observed in the brain, muscle, and gills of wild sea bass (*Dicentrarchus labrax*), Atlantic horse mackerel (*Trachurus trachurus*), and Atlantic chub mackerel (*Scomber colias*) contaminated with MPs with respect to non-contaminated fishes [63]. The LPO increase was also observed by other authors in different species [64,65,66].

Many studies analyse the effect of MPs/NPs exposure on gene expression and the activity of glutathione (GSH) peptide and antioxidant enzymes, mainly CAT, SOD, glutathione peroxidase (GPx), peroxidase (POD), glutathione reductase (GR), and glutathione S-transferase (GST), a biotransformation enzyme [67]. A significant increase in GST enzymatic activity was described in the liver of striped red mullet [68]. In in vivo experimental models, several authors observed MP/NP-induced oxidative damage in various animal species. PS increased ROS production in marine copepod (*Tigriopus japonicus*) exposed to 50 nm and 10 µm microbeads, modifying the expression of genes related to OS (e.g., *Gr, Gst*, *CuZnSod*, and *MnSod*) [57]. Interestingly, the treatment with the antioxidant N-acetyl-L-cysteine (NAC) at a 0.5 mM concentration significantly decreased ROS production. Murano et al. [69] demonstrated an increase in ROS and reactive nitrogen species (RNS) in coelomocytes of sea urchins exposed to 10 and 45 μm PS. An increased total oxidant status and alteration in total antioxidant capacity and LPO were found in the digestive glands of mussels exposed for 96 h to nano-PS particles [70].

Qiao et al. [51] evidenced inflammation and OS in the gut of zebrafish exposed to PS (50 and 500 µg L^−1^ for 21 days) with increased levels of CAT, SOD, GSH, bowel permeability, and dysbiosis. Notably, chronic inflammation is strongly related to OS, gut dysbiosis, and metabolic disturbances [71,72]. In the liver of the same fish species, exposed to the same plastic particles (20–2000 µg L^−1^ for 7 days), tissue inflammation was observed, dose-dependently, and an increase in SOD and CAT enzymes. These alterations were related to an impairment in lipid and energy metabolism [60]. PS also reduced the CAT activity, GSH and ascorbate levels, and increased some mediators linked to OS, evidencing the alteration in the antioxidant mechanisms in zebrafish larvae [61]. Testing the same type of particles, Yu et al. [73] observed in the crab a reduced expression of genes involved in the mitogen-activated protein kinase (MAPK) signalling pathway (*ERK, AKT*, and *MEK*), leading to OS and inflammation, affecting cell survival. Other authors examined the ROS content and the modification of the MAPK-HIF-1/NF-kB pathway in addition to the antioxidant gene expression and enzyme activity in microcrustacean *Daphnia pulex* exposed to nano-PS particles [74]. The low concentrations of NPs (0.1 and 0.5 mg L^−1^) induced low levels of ROS, subsequent activation of the MAPK-HIF-1/NF-kB pathway, and increased antioxidant gene expression and enzyme activities. The highest NPs concentration (2 mg L^−1^) induced the overproduction of ROS, resulting in OS that inhibited growth and reproduction via cellular damage. Tang et al. [75] observed an impairment in the detoxification processes mediated by c-Jun N-terminal kinase (JNK) and extracellular signal-regulated kinase (ERK) signalling pathway activation.

Polyvinyl chloride (PVC) reduced, in a time-dependent manner, the SOD, GPx, and CAT activities in the liver of catfish and augmented LPO in a concentration-dependent manner [65]. Hamed et al. [64] evidenced an increase in total peroxides, as well as LPO, and SOD and CAT activity in *Nile tilapia* sub-chronically exposed to MPs (1–100 mg L^−1^ for 15 days). Interestingly, after a recovery period of 15 days, these alterations were reversed for the fishes exposed to the lowest MP concentration.

Wang et al. [76] evidenced in the digestive glands of mussels exposed to PS (for 14 days and two different water pH conditions) a slight OS; this effect was reversed after a recovery period of 7 days under normal conditions without stressors.

Regarding terrestrial fauna, PE and PS (0–20% d.w.) significantly increased the activity of detoxifying enzymes (POD and CAT) but reduced that of SOD and GST in earthworms (*Eisenia fetida*), using MP amounts that generally do not occur in natural conditions [77]; similar results and conclusions were reported by Rodriguez-Seijo et al. [78]. Conversely, Sun et al. [79] did not evidence a significant modification of the antioxidant defence system and MDA content in earthworms exposed both at low and high MP concentrations (300 and 3000 mg kg^−1^) in soil; similar conclusions were reached by De Felice et al. [80] for the giant snail (*Achatina reticulata*) receiving a diet containing PE-terephthalate. On the other hand, the exposure to MPs modified the oxidative response in the liver, gut, and kidney of mice with increased GPx and SOD and decreased CAT. Substantial alterations were also observed in metabolites related to OS (pyruvate, lysine, and threonine) [52].

*Invitro* experimental conditions, it has been observed that ultrafine PS particles (78 nm^−1^ µm) may enter in pig’s lung and human red blood cells by diffusion or adhesive interactions. PS did not bind the cell membrane but directly accessed to the intracellular environment [81]. Cerebral and epithelial human cells as well as human colon adenocarcinoma Caco-2 cells exposed to the same type of plastic particles (10 mg mL^−1^ for 24 h [82] and 0.1 µm and 5 µm for 12 h [83,84], respectively) boosted ROS accumulation. NH_2_-labeled PS (5–40 μg mL^−1^) enhanced ROS production also in macrophages and lung epithelial cells treated for 6 h and this effect was reduced by a pre-treatment of 1 h with 5 mM NAC [85]. ROS generation was also increased in blood and immune system cells by PP particles (about 25 μm at a concentration of 1000 μg mL^−1^) [33], as well as in human fibroblasts exposed to 100 nm PS (5 µg mL^−1^) [49]; this effect was reduced when the cells were co-exposed to an antioxidant saffron (*Crocus sativus* L.) extract. Dong et al. [34] observed not only increased ROS production but also an amplified expression of heme oxygenase-1 in human lung epithelial cells exposed for 20 min or 24 h to PS (1000 μg cm^2^) [86]. As well known, heme oxygenase-1 is a ubiquitous protein, activated by most of OS inducers. Moreover, renal primary leucocytes from sea bream head exposed to PVC and PE (100 mg mL^−1^ for 1 and 24 h) showed the upregulation of *nrf2* transcription, a gene involved in the response pathway to oxidative stimulus [48]; similar evidence was reported for rainbow trout liver [87].

OS has also been considered among the underlying mechanisms responsible for respiratory system injury provoked by inhaled MPs/NPs in humans (ROS production, cell damage, release of inflammatory mediators) [17,88,89]. Very recently, the bioremediation of OS and hematobiochemical alterations by active components from herbal plants (i.e., lycopene, citric acid, and chlorella) have been investigated in African catfish exposed to MPs (500 mg kg^−1^ diet) [90]. Moreover, Shengchen et al. [91] evidenced the role of NAC in the reduction of ROS overproduction and the imbalance between myogenic differentiation and adipogenic differentiation in mouse myoblasts treated with PS (1–10 µm and 50–100 µm).

Summarized evidence about aquatic, terrestrial fauna, and in vitro models suggests that OS may be considered as one of the key mechanisms determining the toxicity of MPs/NPs. [21,40]. These pollutants induce an inflammatory response leading to ROS and RNS overproduction [56], and often accompanied by a failure of the antioxidant defence system. However, in a few papers, ROS overproduction and an alteration in antioxidant capacity were not observed. A reversal of the effect has been even evidenced after a recovery period. A mitigation effect of antioxidant substances has been observed in in vitro and in vivo studies on aquatic fishes.

## 3. Effects of Micro/Nanoplastics on Reproduction

An updated summary of the studies that focus on MPs/NPs reproductive toxicity is provided in Table 1.

It has been proved that MPs/NPs (70 nm–45 µm) after absorption and translocation from the gut reach various organs, including the gonads (see [7,11,24,25,69,92,93] and references therein). However, because of the blood–gonad barrier, access of larger MPs can be blocked and only the smaller NPs can accumulate [7]. NPs (240 nm) can also accumulate in several reproductive tissues, for instance, in human placenta [94], altering the integrity of the reproductive organs and causing their dysfunction (i.e., defects in the ovary, spermatogenesis, and sperm quality) [23,24,25,95,96]. The presence of PS particles, inversely related to MPs size, was observed in the gonads of sea urchins [69]. Sea urchins were placed in experimental glass tanks (1 specimen per litre) and exposed for 72 h to PS (10 and 45 µm, 10 particles mL^−1^). After extraction by fresh tissue, the PS amount in several organs and gonads was measured by optical microscopy and was inversely related to MPs size [69].

Among the consequences of exposure to plastic debris, reproductive toxicity must be considered [97,98]. In fact, recent data show that MPs/NPs toxicity mechanisms, such as membrane damage, inflammation and immune response impairment [99], genotoxicity, and OS [100], underly the alterations in reproduction in the exposed species. MPs/NPs reduce fecundity up to infertility in male and female organisms [101,102,103] and induce a smaller survival rate of progeny [104], causing impaired reproductive performance.

Most data in the literature on MP/NP-induced reproductive toxicity have been obtained from aquatic species at different trophic levels, mainly focusing on zooplankton. The prolonged exposure of the pelagic copepod *Calanus helgolandicus* to PS beads (20 μm, 75 MPs mL^−1^) causes the production of smaller eggs with reduced hatching success [101]. Similarly, it was shown a decreased fecundity in the copepod *Tigriopus japonicus* exposed to PS (0.5 and 6 μm, 0.125, 1.25, 12.5, and 25 μg mL^−1^) as evidenced by the lessened number of nauplius/female [102]. In the same species exposed to PE and polyamide (PA) (12.5 mg L^−1^), it was observed a longer development time and increased interval time between egg sacs [105].

The exposure of the waterflea *Ceriodaphnia dubia* to PE beads (62.5–2000 µg L^−1^) and fibres (31.25–1000 µg L^−1^) for 8 days determines a reproductive injury with a dose-dependent decrease in both the number and body size of neonates, mainly for the fibres [106]. Moreover, a 40% mortality rate of adults was observed for both types of MPs.

Martins and Guilhermino [107], in a transgenerational study (F0–F3), showed that 21-day exposure of *Daphnia magna* to MPs (100 μg L^−1^) determines adverse impact on growth and reproduction, increasing parental death (up to the extinction of MPs-exposed F1 generation). Moreover, a reduction in the reproduction and population growth rate was observed. The authors showed a slight recovery of these alterations, which needed several generations to occur. Behind neonatal malformations, decreased progeny number and body size were also evidenced by Besseling et al. [97] after *Daphnia magna* exposure to nano-PS (0.22–150 mg L^−1^ for 21 days). Altered reproduction was evidenced in the same small planktonic crustacean after the same time of exposure to 1 and 5 μm MPs (0.012 and 12 mg L^−1^), in addition to an increased time of first brood emission (49%) and usually fewer released clutches (71%) at 12 mg L^−1^ MPs. Other authors observed that the total number of progenies was reduced while the development of immobile juveniles was increased by MPs [104].

In the marine worm *Arenicola marina*, an MP-induced reduction in lipid reserves and available energy was related to reproduction disorders, and suppressed feeding activity [108]. Concentrations of coarse PP fibres (1 mm length), similar to those measured in the environment, induced a negative impact on reproduction in sand crabs (*Emerita analoga*) after about 10 weeks of exposure [109]. In fact, the decreased retention of egg clutches in later embryonic stages and the augmented number of these stages were observed as depending on the amount of the absorbed fibres.

Two months of exposure of the oyster *Pinctada margaritifera* to 6–10 μm PS (0.25, 2.5, and 25 μg L^−1^) was correlated to energy deficit and impaired male gametogenesis, as evidenced by histological analyses [110]. Previously, other authors observed a decrease in oocyte number and diameter, sperm speed, and larval development in adult Pacific oysters (*Crassostrea gigas*) exposed to PS (23 μg L^−1^) for the same exposure time [103]. Moreover, nano-PS decreased the oyster fertilization efficacy and early life-stages (i.e., embryogenesis and larval growth), with several alterations up to the developmental arrest. The severity of these modifications depended on the size and structure functionalization, with NH_2_ particles (50 nm) exhibiting the major toxicity on gametes and embryos [111]. More recently, the same authors evidenced that acute exposure of oyster spermatozoa to different concentrations of PS beads induced a high spermiotoxicity, characterized by a decrease in the motility and velocity of spermatozoa [112].

Chisada et al. [113] studied the Japanese aquatic vertebrate medaka (*Oryzias latipes*), showing a decrease in egg number and hatching rate after 3 months of exposure to PE microbeads (10–63 μm, 65 and 650 μg L^−1^). Notably, the growth rate was also reduced at the highest concentration [113]. Similar data were obtained after feeding medaka with a PS-contaminated diet (10 μm, 0.5–2 mg g^−1^), evidencing fewer egg production during the development from the immature stage to adult deposition age [114].

Conversely, Assas et al. [115] did not notice modifications in egg production, growth, and survival of medaka after 3 weeks of exposure to a lower PS concentration (2 μm, 44 μg L^−1^). In the annelid *Lumbriculus variegatus* exposed to PE (concentrations between 0.51 and 20 g kg^−1^ dry sediment), no effect was observed on reproduction and biomass [116]. Moreover, no significant alterations in the reproductive activity were evidenced in the freshwater cnidarian, *Hydra attenuate* [117]. Qiang et al. [118] exposed the zebrafish to micro-PS (1 µm, 10–1000 µg L^−1^ for 21 days) without observing variations in the testosterone and 17-β-estradiol levels. The authors also reported higher mRNA expression of some steroidogenic genes in testis than in ovary, but only at the highest concentration employed, without significant effects on reproduction and progeny development. In a similar study, zebrafish exposure via feeding to nano-PS particles (42 nm, 1 mg g^−1^ bw for 1 week) caused their transfer in the yolk sac, probably through the adsorption onto vitellogenin. However, no effect on offspring survival rate and development was evidenced [119]. Based on this evidence, MPs/NPs can represent a potential hazard for aquatic biota and mainly for species conservation.

Regarding MPs/NPs reproductive toxicity in terrestrial species, the literature data are limited and mainly focused on experimental models by using rodents; to the best of our knowledge, very few papers involve invertebrate species. MPs/NPs present in soil could be ingested by organisms, such as worms, potentially impairing their reproduction. Indeed, the exposure of *Caenorhabditis elegans* to MPs (1–100 mg L^−1^) caused a reduction in offspring, independently from MP type, with a bell-shaped trend [120]. Analogously, Lahive et al. [121] showed a dose-dependent damage in reproductive efficiency, characterized by fewer juveniles per adult when the worm *Enchytraeus crypticus* was exposed to 13–18 μm PA (20, 50, 90 and 120 g nylon kg^−1^ dry soil); however, no effect on survival was observed. Recently, a comparison between the transgenerational reproductive toxicity induced by NPs and NPs-NH_2_ in *Caenorhabditis elegans* was shown. The parental exposure to NPs-NH_2_ determined a stronger transgenerational toxicity on reproductive capacity and gonad development than when exposed to NPs. This effect was mainly due to the induction of germline apoptosis [122].

NPs from PE-MPs breakdown have been experimentally investigated in soil ecosystem using the earthworm *Eisenia andrei* [28]. The authors described the impact of NPs on coelomocyte viability and damage on male reproductive organs, while negligible effects were evidenced on female reproductive organs. They reported the impairment of spermatogenesis and the arrangement of sperm bundles in the seminal vesicles. When springtail *Folsomia candida* was chronically exposed to PE (<500 μm), their reproductive function was severely reduced, up to 70% at the highest concentration tested (1% PE *w*/*w* in dry soil) [123].

Reported results indicate that MPs/NPs, mainly introduced through diet, can accumulate in male gonads, affecting reproduction in aquatic and terrestrial species, with a transgenerational impact (e.g., in *Daphnia magna* and *Caenorhabditis elegans*). MPs/NPs exert their adverse effect mainly on gametogenesis, embryos, and progeny. Among the main mechanisms involved, we can include the altered metabolism of sex hormones, inflammatory process, OS, and disruption in energy balance. Reproductive and developmental toxicity has been investigated in males, but less frequently in female organisms; thus, rarely analysing the MPs/NPs female gonadal content and without evidence of particle presence. In females, MPs/NPs toxicity also may be related to the unbalance in sex hormone synthesis [100].

Very recently, adverse effects on the reproductive system were highlighted in male mice exposed for 35 days to 5 µm PS (approximatively 0.65, 6.5 and 65 µg day^−1^/mouse in drinking water), with a reduced spermatids/spermatozoa quantity and relevant sperm malformations at the highest doses [124]. Increased expression of NF-kB transcription and inflammatory cytokines interleukin (IL)-1β and IL-6 was evidenced and related to sperm alterations [124]. Similar results were obtained by Jin et al. [25] in mice exposed daily for 28 days to PS (0.5, 4, 10 µm) by gavage using a thousand-time higher dose than that used by Hou et al. [124]. The authors also evidenced a decreased testosterone level and damaged blood–testis barrier.

It was also reported that the daily exposure of male mice (*Mus musculus*, CD-1) to PE (0.4–5 µm, 100 mg kg^−1^ bw, for 30 days) by oral gavage resulted in a decreased sperm number and an increased level of the biomarker acid phosphatase (ACP) [24]. This enzyme is mainly located in Sertoli cells and provides structural and nutritional support in spermatogenesis. Likewise, alterations in sex hormones and a reduction in sperm quality standards (i.e., spermatozoa concentration, motility, morphology, and DNA integrity) were detected in Wistar rats following oral nano-PS exposure (1, 3, 6 and 10 mg kg^−1^ bw day^−1^, for 35 days) [95]. The presence of small PS particles (few µm and nm) was detected in BALB/c mice [25] and Wistar and Fisher 344 rat testes by using biofluorescence imaging systems [95,125], and PE accumulation was also detected in mice (*Mus musculus*, CD-1) by gas chromatography–mass spectrometry [24]. The daily administration of PE (roughly 3.75, 15, or 60 mg kg^−1^ bw by gavage) to ICR mice for 3 months determined a marked enlargement of the Fallopian tubes in the parent mice and a strong injury on reproduction [126]. At the highest MP dose, a significant reduction in the number of live births per dam and body weight of pups was evidenced; an alteration of the sex ratio (male/female) of the progeny was also shown. All these studies on murine models have utilized high MP concentrations, which are rarely detected in environmental matrices [109,118].

Recently, pregnant rats were exposed to labelled nano-PS beads (20 nm) via intratracheal instillation (at gestational day 19) [127]. The authors observed that maternal pulmonary exposure to PS results in the translocation of PS particles to placental and foetal tissues making the fetoplacental unit vulnerable to deriving adverse effects.

The occurrence of MPs/NPs in human gonads and their possible effects on reproduction have not been so far identified. However, the presence of plasticizers, including phthalates in human semen, was found, and negatively correlated to sperm quality and concentration [128,129]. Pigmented MPs have been found in placental tissues (maternal and foetal portion and chorioamniotic membrane) obtained from women with normal pregnancies [94], but the consequences on foetuses have been overlooked. PS (up to 240 nm) is known to cross the human placenta, as demonstrated by an ex vivo perfusion model [130]. Conversely, Hesler et al. [131] did not evidence placental translocation, using in vitro models. However, the same authors observed a slight embryotoxic effect and non-genotoxic damage. Nano- and micro-PS internalization in placenta supports the hypothesis of MPs/NPs accumulation and the following impairment of the placental barrier function. Despite being limited, these data open a new scenario to understand the reproductive toxicity induced by these pollutants and the risk for human health.

Controversial data on toxic reproductive effects can be explained by different MPs/NPs concentrations, size, or the surface functionalization used in the several experimental procedures. NPs, due to their smaller size, could possibly induce effects at different biologic levels when compared to MPs. Conversely, Hesler et al. [131] observed an increased toxicity when murine fibroblasts and embryonic stem cells cultures were treated with micro-PS particles compared to nano-PS particles.

Both MPs and NPs determine gamete abnormalities, but NPs can also alter membrane fluidity, hampering contact between gametes. Particle size can differently affect embryo development. MPs cannot penetrate the embryo, but by covering the surface of the chorion, they prevent oxygen uptake, with serious consequences for embryo health and hatching. On the other hand, NPs, being able to enter the embryo, accumulate in the yolk sac, altering nutrient absorption (see [93] and references therein). Even if evidence collected on terrestrial mammals and human placenta accumulation are still preliminary, they appear intriguing and open a new scenario to understand the reproductive toxicity and evaluate the risk for human health induced by these pollutants.

### Effects of the Co-Exposure to Other Pollutants on Reproductive Toxicity by Micro/Nanoplastics

MPs can sensitize living organisms to environmental factors that may impact fertility during the lifespan, intensifying the toxic effect of environmental pollutants or endocrine disruptors on reproduction. To date, the role of the combined exposure to MPs/NPs and other chemical pollutants on reproductive toxicity has been rarely investigated. Cheng et al. [132] reported an increase of annetocin mRNA expression in earthworms (*Eisenia fetida*) exposed to MPs and atrazine with respect to each pollutant alone. Annetocin is considered a regulating gene involved in the reproduction process of soil earthworms.

In African catfish, the exposure for 96 h to low-density PE particles loaded with phenanthrene (Phe) (50, 500 μg L^−1^ MP and 10, 100 μg L^−1^ Phe) determined an inverse U-shaped pattern of *GnRH* and *ftz-f1* mRNA, with a significant downregulation effect for *GnRH* (at 10 μg L^−1^ Phe combined with 500 μg L^−1^ MP) with respect to virgin MP in the brain [133]. *GnRH* and *ftz-f1* are genes involved in the biosynthesis and balance of steroid hormones; therefore, their altered transcription may impact the reproductive process.

Alterations in different reproductive parameters were reported by Pacheco et al. [104] in *Daphnia magna* sub-chronically exposed to a mixture of gold nanoparticles (0.2, 2 mg L^−1^) and MPs (0.02, 0.2 mg L^−1^). The combined exposures determined a further detrimental effect on the decreased total number of offspring (equivalent to the sum of mobile juveniles, immobile juveniles, and aborted eggs) and augmented the total number of aborted eggs caused by gold nanoparticles alone.

**Table 1:** Toxic effects of MPs/NPs on the reproduction of aquatic and terrestrial organisms.

Species	MPs/NPs Type	MPs/NPs Size	MPs/NPs Shape	Exposure Time	Effects on Reproduction	Reference
*Arenicola* *marina*	UPVC	130 µm	Beads	28 d	Alteration of growth, reproduction and survival related to suppressed feeding activity and reduced energy reserves.	[108]
*Calanus* *helgolandicus*	PS	20 µm	Beads	9 d	Smaller eggs with reduced hatching success.	[101]
*Carcinus* *maenas*	PS	0.5 µm	Beads	1 h–21 d	Presence of MPs in ovaries.	[11]
*Ceriodaphnia dubia*	PE	1–4 μm	Beads, fibres	8 d	Augmented mortality rate. Reduction of offspring number and body size.	[106]
*Clarias* *gariepinus*	LDPE	<60 μm	Irregular shape	96 h	Down-regulation of genes involved in steroid hormones biosynthesis.	[133]
*Crassostrea* *gigas*	PS	50 nm	Beads	1 h	Decreased percentage of motile spermatozoa and velocity. Reduced embryogenic success. No significant effects on morphology and functional characteristics of spermatozoa.	[112]
	PS	2 μm, 50, 500 nm	Beads	1.5 h, 36 h	Decreased fertilization success and embryo-larval development depending on particle functionalization.	[111]
	PS	2, 6 μm	Beads	60 d	Decrease in oocyte number, sperm diameter and speed. Reduction of larval development.	[103]
*Danio rerio*	PS	1 µm	Beads	21 d	Higher expression of steroidogenic genes in testis but not in ovaries. No variation of testosterone and 17-β-estradiol levels. No significant effects on progeny development.	[118]
	PS	70 nm	Beads	30 d	Accumulation of MPs in gonads.	[7]
*Daphnia magna*	MPs	1–5 µm	Beads	21 d	Parental death up to the extinction of F1 generation. Reduced fecundity and population growth rate. Slight transgenerational recovery after the depuration period.	[107]
	MPs	1–5 µm	Beads	21 d	Increased time of first brood emission. Increased number of immobile juveniles. Decreased clutches and number of progenies. Worsened effects with the co-exposure to gold nanoparticles and MPs.	[104]
	PS	70 nm	Beads	21 d	Impairment of population growth. Reduction of progeny. Decrease in newborn number and body size. Increase of progeny malformations.	[107]
*Emerita analoga*	PP	1 mm	Fibres	71 d	Decrease in retention time of egg clutches. Augmented number of later embryonic stages.	[109]
*Hemicentrotus pulcherrimus*	wild MPs	27–4742 μm	Fibres, fragments, sheets, beads	u	Presence of MPs in gonads.	[92]
*Hydra attenuate*	PE	<400 µm	Irregular shape	30 m, 60 m	No significant impairment of reproduction.	[117]
*Mytilus edulis*	PS	0.5 µm	Beads	1 h–21 d	Presence of MPs in ovaries.	[11]
*Oryzias* *javanicus* *Oryzias latipes*	PS	2 µm	Beads	21 d	No alteration in growth, survival and egg production.	[115]
*Oryzias latipes*	PS	10 µm	Beads	70 d	Reduction in egg production.	[114]
	PE	10–63 µm	Beads	90 d	Fewer egg number, hatching rate and growth rate.	[113]
*Paracentrotus lividus*	PS	10, 45 μm	Beads	72 h	Presence of MPs in gonads.	[69]
*Pinctada* *margaritifera*	PS	6, 10 μm	Beads	60 d	Impaired gametogenesis. Histological alterations in the gonads.	[110]
*Tigriopus* *japonicus*	PE PA	10–30 µm 5–20 µm	Irregular shape	24 h, 14 d	Prolongation in development time and in interval time between egg sacs.	[105]
	PS	0.5, 6 µm	Beads	96 h	Impaired fecundity evidenced by the reduction in number of nauplius per female.	[102]
*Caenorhabditis elegans*	PS	35 nm	Beads	4 d	Transgenerational effects on reproductive function, gonadal development and germline apoptosis, depending on particle functionalization.	[122]
	LDPE PLA/PBAT	57 µm 41 µm	Irregular shape	6 d	Reduction in offspring.	[120]
*Eisenia andrei*	PE	180–212 μm 250–300 μm	Beads	21 d	Impaired spermatogenesis and histological alterations in male gonads. Negligible effects on oogenesis and female gonads.	[28]
*Enchytraeus crypticus*	PA	13–18 μm 63–90 μm 90–150 μm	Irregular shape	20 h	Reduction of juveniles per adult.	[121]
*Folsomia* *candida*	PE	<500 µm	Beads	28 d	Decreased reproductive function with reduction of juvenile number.	[123]
BALB/c mice	PS	0.5, 4, 10 μm	u	28 d	Presence of PS into testicular cells. Decreased sperm quality and increased abnormality rate. Reduced testosterone levels. Testicular inflammation and damaged blood-testis barrier.	[25]
ICR mice	PS	5 μm	u	35 d	Decreased number of spermatids/spermatozoa with altered sperm quality. Increased testicular inflammation and apoptosis rate.	[124]
	PE	40–48 μm	u	90 d	Enlargement of Fallopian tubes in dams. Fewer live births per dam and altered sex ratio of progeny. Reduced body weight of pups.	[126]
Sprague Dawley rats	PS	20 nm	Beads	24 h	PS particles translocation to placental and foetal tissues 24 h after maternal exposure.	[127]
Wistar rats	PS	25, 50 nm	Beads	35 d	Presence of PS in testis. Histological alterations of testicular tissue. Alteration of sex hormones levels. Impaired spermatogenesis and increased DNA damage.	[95]
Human placenta	MPs	u	Beads, irregular shape	u	Presence of MPs fragments in human placental tissues.	[94]
	PS	0.5 µm, 50 nm	Beads	24 h	Internalization of PS particles in placental cells.	[131]
	PS	50, 80, 240, 500 nm	Beads	3 h	Crossing of the placental barrier by PS particles in a size-dependent manner.	[130]

d: days; h: hours; LDPE: low-density polyethylene; m: minutes; MPs: microplastics; NPs: nanoplastics; PA: polyamide; PE: polyethylene; PLA/PBAT: polylactide/poly(butylene adipate-co-terephthalate); PP: polypropylene; PS: polystyrene; PVC: polyvinyl chloride; u: unknown; UPVC: unplasticised polyvinylchloride.

## 4. Role of Oxidative Stress on Micro/Nanoplastic-Induced Reproduction Alterations

The impairment of MPs/NPs on reproduction can be caused by the redox unbalance since OS is fully recognised as a key factor in ovarian and testicular dysfunctions and development of infertility [134,135]. The main toxic effects of MPs/NPs on reproduction via OS are summarised in Table 2.

### 4.1. Reproductive Toxicity of Micro/Nanoplastics Mediated by Oxidative Stress in Aquatic Organisms

To the best of our knowledge, the above toxicity mechanism underlying reproductive alterations was observed only in aquatic species experimentally exposed to MPs/NPs, and not in wild animals. In these experimental models, OS was caused by the increased ROS/RNS production [136] and the reduction in antioxidant defences [100].

Firstly, the marine copepod *Paracyclopina nana* acutely exposed to PS (0.05, 0.5 and 6 µm, 20 µg mL^−1^) showed an increase in both ROS production and antioxidant enzyme activities (SOD, GR, GPx and GST) [58]. These effects were inversely related to PS size. ROS-dependent activation of the MAPK pathway and augmented expression of inflammatory mediators, i.e., p-ERK, p-p38 and Nrf2, resulted in the defence response to OS. The latter was related to the impairment of reproductive function with fewer newborn nauplii [58]. In comparable experimental conditions, in the monogonont rotifer *Brachionus koreanus*, similar results were reported of ROS overproduction, as well as an increase in antioxidant enzyme activity and p-p38 and p-JNK [59]. These effects were related to a reduction in fecundity and lifespan, as well as a prolongation of reproduction time. Interestingly, in these studies, ROS scavenger NAC (0.5 mM) reduced the ROS production. Xue et al. [137] exposed the freshwater rotifer *Brachionus calyciflorus* to PE (0.5 × 10^3^–1.25 × 10^4^ particles mL^−1^, 10-22 µm) and observed an oxidative unbalance proved by abnormal SOD and GPx activities. The authors showed a compromised reproductive capability (alterations of survival, lifespan, rates and time of reproduction, and population growth), linking these effects directly to OS and indirectly to a reduced energy availability necessary to counteract the oxidative process. Moreover, impaired fecundity was revealed in *Daphnia pulex* exposed to PS (75 nm, 0–2 mg L^−1^, for 21 days), showing a prolonged time to first eggs and to first clutch, and a reduction in number of clutches, newborns per clutch, and total number of neonates per female [138]. The authors observed an altered gene expression of antioxidant enzymes (*SOD, CAT, GST*, and *GPx*), with a non-linear trend, and an augmented expression of *HSP70* and *HSP90* genes. As known, heat shock proteins are considered biomarkers of stress induced by chemical pollutants and contribute to maintaining the protein composition unchanged [139]. Very recently, the effect of the same spherical PS nanoparticles on reproduction of *Daphnia pulex* was studied, by examining the proteome together with molecular and biochemical data involved in adverse outcome occurrence, evidencing ROS production and thus OS [140,141]. Both subacute and chronic NPs exposure negatively affected cumulative offspring production. Modifications of specific immune and metabolic signalling pathways and cellular metabolism were observed, resulting in growth inhibition and decreased reproduction. Previously, these authors reported that NPs at typical environmental concentrations (1µg L^−1^) have potent long-term toxic effects on *Daphnia pulex* [142]. Chronic exposure to NPs modulated the response of antioxidant defences, vitellogenin synthesis, development, and energy production in the F0–F1 generations. Conversely, in the F2 generation, NPs did not affect the survival but altered the growth rate and reproduction, showing inhibitory effects on antioxidant responses.

Lee et al. [66] showed that 96 h treatment of the marine mysid (*Neomysis awatschensis*) with PS (5 × 10^5^ particles mL^−1^, 1 and 10 µm) determined an increase in the MDA levels and antioxidant enzymes (SOD, CAT, GR, GSH, and GPx) activity in organisms exposed both to 7 and 30 days after hatching. The results did not follow a clear linear trend, influenced by particle size, exposure time, and mysid age. Moreover, the protracted exposure caused a decrease in the 20-hydroxyecdysone levels (a steroid-like hormone strictly involved in arthropod moulting and growth, as well as ovarian and sexual development) and total newborns per female, more evident at the highest concentration used. Recently, Qiang and Cheng [136] exposed zebrafish sub-chronically to 1 µm PS, and observed increased ROS generation in both the ovary and testis (at 100 and 1000 µg L^−1^ concentrations), as well as an augmented spermatocytes apoptosis rate (at 1000 µg L^−1^). Moreover, an apoptosis increase (i.e., p-53, Bax, and caspase-7-8-9) and thickness reduction in the basement membrane were observed in male gonads. In the same species, a long-term exposure (35 days) to PS-MPs caused an alteration in the metabolic mechanisms and gene regulation patterns induced by ROS generation and OS [143].

A broken redox balance was also found in marine medaka *Oryzias melastigma* after exposure to environmentally relevant concentrations of PS (2–200 µg L^−1^, 10 µm, for 60 days) [100]. The results showed an increase in MDA levels and a decrease in antioxidant enzyme activity (SOD, CAT, GST, GPx, and GSH) in the ovary and testis, related to histological changes (i.e., impaired seminiferous lobules architecture, reduction in sperm fluid in male, reduced number of mature spawning follicles, and augmented presence of early vitellogenic oocytes in female). Moreover, the authors evidenced a reduction in plasmatic 17-β-estradiol and testosterone in females related to alteration of the hypothalamic–pituitary–gonadal axis. The latter effect determined the gonadosomatic index reduction, as well as the belated gonadal development. The reproductive impairment was also demonstrated by the onset of transgenerational consequences (i.e., decrease in the fertility and hatching rate of progeny). Thereafter, the same authors adopted a whole life-cycle exposure of medaka to determine the impact of PS on the hatching of embryos, growth and reproduction of the F0 generation, and embryonic and larval development of the F1 offspring [144]. Interestingly, 150 days of exposure caused gonad damage and a decreased egg production and fertilization rate. Transcriptome analysis evidenced modifications in steroid hormone biosynthesis and cytochrome P450 pathways in the testes of male fish after 20 μg L^−1^ MPs exposure.

Differently from the previous evidence, another transgenerational study revealed a scarce involvement of an antioxidant defence system in specimens of *Danio rerio* orally exposed to nano-PS (1 mg g^−1^ bw, for 1 week). No alteration in parental fertility and offspring development was observed, except a reduction in GR in the F0 male gonads, as well as in the F1 larvae arising from exposed parents [119].

The reported studies show that MPs/NPs determine ROS overproduction, and where the related OS induction activate the signalling pathways involved in the production of inflammatory mediators, causing immune and endocrine alterations. These effects result in decreased fecundity, impaired reproductive capability, and compromised growth and survival of newborns, observed both in zooplankton and aquatic species at higher trophic levels. The mitigation effect of antioxidant substances has been very poorly investigated.

### 4.2. Reproductive Toxicity of Micro/Nanoplastics Mediated by Oxidative Stress in Terrestrial Organisms

To date, few papers have analysed reproductive disorders related to MP/NP-induced OS in terrestrial organisms. Lei et al. [145] observed a reduced number of embryos and clutch size in nematode *Caenorhabditis elegans* acutely exposed to 5 mg m^−2^ PA, PE, PP, PVC (70 µm) and PS (0.1, 1, and 5 µm). The authors also showed increased expression of GST-4 enzyme in the gut, suggesting the involvement of OS. Other authors reported the enhancement of ROS production in the same species acutely exposed to 50 nm PS (86.8 mg L^−1^) [146] or 1 µm PS (1, 10 and 100 µg L^−1^) [147]; in both cases, a reduced number of newborns was evidenced. Moreover, the OS involvement was further assessed by the increased expression of OS-related genes (i.e., *gst-4* and *sod*) and lipofuscin levels, whose accumulation is an indicator of cellular toxicity in response to oxidative damage [147].

The exposure of *Caenorhabditis elegans* to PS-NPs (20 and 100 nm) determined a severe transgenerational toxicity more evident for the smaller sizes (detected at the F1–F6 generations) [148], and the toxicity on reproduction and locomotion behaviour was associated to increased ROS production.

Very few and recent studies have been performed on rodent experimental models showing effects comparable to those observed in aquatic species. The dose-dependent increase in ROS and MDA content and the decrease in GSH activity were evidenced in mice testis after chronic oral exposure to PS (5–5.9 µm, 0.01–1 mg day^−1^) [149]. In testis, a redox unbalance resulted in a not always linear activation of the JNK and p38 MAPK pathways and subsequent release of pro-inflammatory cytokines (TNF-α, IL-1β and IL-6) and pro-apoptotic caspase-3. The same authors also evidenced a reduction in sperm cell number and quality, in testosterone levels, and in succinate dehydrogenase (SDH) and lactate dehydrogenase (LDH), enzymes involved in sperm generation and energy metabolism. Interestingly enough, the above effects were mitigated by the co-administration of the antioxidant NAC (100 mg kg^−1^ bw day^−1^ intraperitoneally), further supporting the key role of OS in the onset of reproductive damage [149].

Deng et al. [24] showed the presence of MPs in the Sertoli cells of mice exposed to PE (0.4–5 µm, 100 mg kg^−1^ bw for 30 days) and an increase in the SOD and MDA content in testis. The authors evidenced a reduction in SDH and the augmentation of ACP and LDH, compromised spermatogenesis, as well as reduced number and viability of sperm cells and testis weight. Similarly, Ijaz et al. [150] reported reduced sperm count, motility, and viability in rats treated with PS (2–2000 μg L^−1^). The effects, more evident at higher concentrations, were accompanied by a decreased expression of antioxidant enzymes together with an increase in LPO and ROS. Moreover, the plasma levels of a few steroidogenic enzymes and the key hormones of male reproductive health were reduced. The authors pointed at OS to explain the effects reported above and the histological damages in the testis.

Li et al. [151] studied the effect of PS (0.015–1.5 mg day^−1^ for 90 days) on the blood–testis barrier impairment in Wistar rats, leading to the damage of the seminiferous tubules, apoptosis of spermatogenic cells, and decreased sperm motility and concentration. PS induced OS (increasing MDA and decreasing antioxidant enzymes) and activated the p38 MAPK pathway, depleting Nrf2. Analogously, higher doses of PS (4 and 10 μm, 20 and 40 mg kg^−1^ bw) induced an alteration in the blood–testis barrier and, consequently, spermatogenesis dysfunction in BALB/c mice, evidenced by cytoskeleton disorganization [152]. This effect strictly depended on the increased ROS production that causes the imbalance in mTOR1 and mTOR2, which plays a role in regulating the actin cytoskeleton.

The effects of 0.5 µm PS on ovary were also evaluated by An et al. [23] treating female rats with 0.015–1.5 mg day^−1^ MP in drinking water for 3 months. The authors evidenced the presence of PS particles in the ovary and the induction of a fibrotic process limited by a pre-treatment with 3 mmol NAC for 4 h.

Moreover, granulosa cells (GCs) from untreated rats were incubated with 1–25 μg mL^−1^ PS for 24 h; increased ROS and MDA content was observed, as well as decreased SOD, CAT, and GPx activities, evidencing the pivotal role of OS in the mechanism of toxicity of MPs. The increase in apoptotic rate of GCs was related to a Bax increase and Bcl-2 reduction, evidencing the activation of the Wnt/β-catenin pathway in the ovary. Lastly, the authors noticed the alterations in follicle morphology and number in the ovary, and the decrease in the anti-Müllerian hormone (AMH) [23].

**Figure 1:**
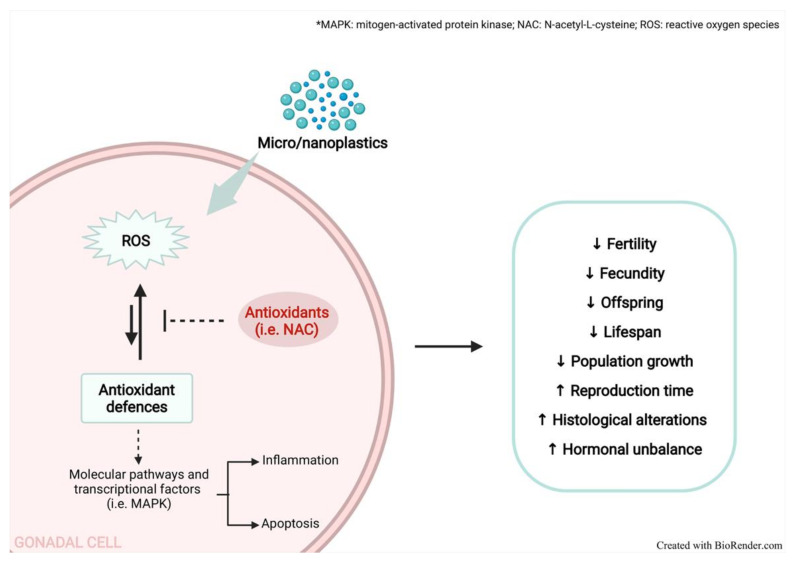
Oxidative stress-mediated effects of MPs/NPs on reproduction.

Accordingly, Hou et al. [96], in the same experimental conditions, observed overlapping results regarding the antioxidant enzymes, MDA, and AMH level in ovary tissue. Moreover, the authors hypothesized that OS may trigger the inflammation-associated factor NLRP3 inflammasome/caspase-1 signalling pathway, leading to pyroptosis and apoptosis of GCs, suggesting the involvement of PS in female infertility. The main OS-mediated reproductive alterations caused by MPs/NPs are shown in Figure 1.

In terrestrial species, similarly to aquatic species, the studies reviewed show that MPs/NPs affect reproduction, determining ROS generation, MDA production, and the increased expression of OS-related genes. The latter effect is responsible for the release of pro-inflammatory cytokines and pro-apoptotic mediators that cause impaired spermatogenesis, reduced sperm, and GCs viability. The apoptosis of sperm cells is also a consequence of the blood–testis barrier damage. The limited number of studies on mammalian species does not allow us to well define the passage of the MPs/NPs in reproductive organs and the following alterations. The beneficial role of antioxidant substances in reducing ROS overproduction remain to be elucidated.

### 4.3. Reproductive Toxicity of Micro/Nanoplastics in Combination with Other Chemical Pollutants Mediated by Oxidative Stress

To the best of our knowledge, only very few papers report data on reproductive toxicity mediated by OS and which are related to joint exposure to MPs/NPs and other xenobiotics. The four-week exposure to MPs and pesticide atrazine in earthworm (*Eisenia fetida*) determined ROS accumulation and MDA production and a decrease in SOD, CAT, and GST activity [132].

Deng et al. [24] showed that phthalates esters (PAEs) may strengthen MP-induced reproductive impairment in mice and hypothesized that the effect is related to OS. Indeed, the exposure to MPs combined with a PAE mixture determined a significant decrease in sperm number and vitality compared to virgin MPs. Testicular weight was also reduced but only at the highest PAE concentration (50 µg L^−1^). The combined treatment induced significant increase in the ACP and LDH levels in the testes and decreased SDH. The mixture with the highest PAE concentration induced an enhancement of the oxidative unbalance with augmented SOD activity and MDA content. Finally, the transcriptomic analysis performed on the left-side testis after the exposure to MPs plus the high di-2-ethylhexylphthalate concentration or the PAE mixture aggravated the toxicity of the MPs. It was observed that the altered expression of genes was involved not only in endocrine and immune system functioning but also in lipid and energy metabolism. The authors speculated that the latter reported alterations may play a role in reproductive stress.

In the rotifer *Brachionus koreanus*, nano-PS pre-exposure followed by 2,2’,4,4’-tetrabromodiphenyl ether or triclosan challenge induced a reduction in the reproductive output compared to single POP exposure [153]. This alteration seems to be associated to the membrane dysfunction provoked by OS, causing impairment of membrane fluidity and permeability.

In the same species, Yoon et al. [154] examined how multiple exposure to tributyltin (TBT)—an organotin compound—MPs, and a dietary regimen affects reproduction and can influence offspring. Surprisingly, the results showed that environmentally relevant MP concentrations and feed can attenuate the TBT toxic effects, evidencing the major efficacy of the diet. However, 24 h of MPs and/or TBT exposure in F0 can modulate the F1 life cycle, changing the population growth. The co-exposure to PS and TBT induced a slight increase in ROS production and alteration in SOD and CAT activities, without a linear trend at different nutritional schemes. MP co-exposure did not exacerbate the effect of decreased fecundity induced by TBT, even if a slight mitigation was observed for the lowest concentration of the microalgae *Tetraselmis suecica* in the diet. Similarly, the ingestion of irregular PS-MPs (exposure level of 6.4–100,000 particles mL^−1^) by the freshwater gastropod *Lymnaea stagnalis* did not induce significant effects on survival, reproduction, energy reserves, and OS, and did not worsen the reproductive toxicity of copper [155]. The lack of adverse effects may be explained by stress resilience or the adaptation of the freshwater gastropod to the contaminants, which occurs not only at environmentally relevant concentrations but also at higher concentrations.

Some authors report that reproductive toxicity via OS worsens with the co-exposure of MPs/NPs and other chemical pollutants. Some others find instead that MPs/NPs may even mitigate the effects of chemical pollutants, thus making it difficult to draw clear conclusions.

**Table 2:** Toxic effects of MPs/NPs on reproduction in aquatic and terrestrial species via OS and oxidative unbalance.

Species	MPs/NPs Type	MPs/NPs Size	MPs/NPs Shape	Exposure Time	Effects on Reproduction	Oxidative Unbalance	Reference
*Brachionus calyciflorus*	PE	10–22 µm	Beads	Until the death of maternal rotifers	Reduction of reproductive parameters, such as: survival, lifespan, rates and time of reproduction and population growth.	↓ SOD ↑ GPx No alteration of MDA levels	[137]
*Brachionus* *koreanus*	PS	0.05 µm	Beads	24 h	Mitigated effects on reproductive toxicity of TBT following the co-exposure to MPs.	↑ ROS Impairment of SOD and CAT activities	[154]
	PS	0.05, 0.5, 6 μm	Beads	24 h	Reduced number of rotifers and fewer newborns following the co-exposure to MPs and POPs.	↑ ROS ↑ MDA	[153]
	PS	0.05, 0.5, 6 μm	Beads	24 h, 12 d	Reduced fecundity and lifespan. Augmented reproduction time.	↑ ROS ↑ SOD, ↑ GR, ↑ GPx and ↑ GST	[59]
*Danio rerio*	PS	1 µm	Beads	21 d	Increased apoptosis rate in testis but not in ovary. Impairment of basement membrane.	↑ ROS in ovary and testis	[136]
	PS	42 nm	Beads	7 d	Transfer in the yolk sac without effects on survival and development of offspring.	↓ GR in male gonads and in F1 larvae	[119]
*Daphnia pulex*	PS	~71 nm	Beads	21 d	Lower production of cumulative offspring.	↑ GSH and ↑ GST	[141]
	PS	~75 nm	Beads	21 d	Reduction of growth rate, total clutches and offspring per female in F2 generation. No significant effect on F0 and F1 generations.	↑ H_2_O_2_, ↑ CAT and ↑GST Affected expression of genes related to oxidative stress (i.e., *CAT, GSTD, MnSO* and *CuZn SOD*)	[142]
	PS	75 nm	Beads	21 d	Decreased number of clutches and newborns. Increased time to first eggs and to first clutch.	Altered gene expression of *SOD, CAT, GST* and *GPx*	[138]
*Eisenia fetida*	LDPE	550–100 μm	Irregular shape	28 d	Increased gene expression of annetocin. Worsened effects following the co-exposure to MPs and atrazine.	↑ ROS, ↑ MDA and ↑ 8-OHdG ↓ SOD, ↓ CAT and ↓ GST	[132]
*Lymnaea* *stagnalis*	PS	<63 µm	Irregular shape	28 d	No effects on the egg numbers and survival rate.	No effect on oxidative balance	[155]
*Neomysis awatschensis*	PS	1, 10 µm	Beads	96 h	Reduction of 20-hydroxyecdysone levels. Fewer newborns per female. No alteration of survival rate of newborns.	↑ MDA ↑ SOD, ↑ CAT, ↑ GR, ↑ GSH and ↑ GPx	[66]
*Oryzias* *melastigma*	PS	2 μm	Beads	150 d	Histological alteration in testis and ovaries. Reduced eggs production and suppressed fertilization rates. Impairment of steroid hormone biosynthesis. Accelerated sexual maturity in female. Premature hatching and altered growth in F1 generation.	↑ MDA in ovary and fertilized eggs ↓ SOD, ↓ CAT, ↓ GPx, and ↓ GST in testis, ovary and fertilized eggs	[144]
	PS	10 μm	Beads	60 d	Histological alteration in male and female gonads. Reduced 17-β-estradiol and testosterone plasmatic levels in female related to gonadosomatic index reduction and delayed gonadal development. Decreased fertility and hatching rate of progeny.	↑ MDA ↓ SOD, ↓ CAT, ↓ GST, ↓ GPx and ↓ GSH in ovary and testis	[100]
*Paracyclopina nana*	PS	0.05, 0.5, 6 μm	Beads	24 h	Fewer newborn nauplii.	↑ ROS ↑ SOD, ↑ GR, ↑ GPx and ↑ GST	[58]
*Caenorhabditis elegans*	PS	1 µm	Beads	72 h	Fewer newborns.	↑ ROS ↑ *gst-4* and ↑ *sod* genes expression ↑ Lipofuscin levels	[147]
	PS	50 nm	Beads	24 h	Fewer newborns per worm.	↑ ROS	[146]
	PA, PE, PP, PVC and PS	70 μm 0.1, 1, 5 µm	Irregular shape and beads	2 d	Fewer embryos and smallest clutch size.	↑ GST-4	[145]
BALB/c male mice	PS	5–5.9 µm	Beads	42 d	Reduced sperm quality and quantity. Decreased testosterone levels. Reduction in SDH and LDH activities.	↑ ROS ↑ MDA ↓ GSH	[149]
*Mus musculus*, male mice	PE	0.4–5 μm	Beads	30 d	Presence of PE in testis. Impaired spermatogenesis evidenced by alteration in ACP, LDH and SDH levels. Reduced sperm quality. Worsened effects and reduced testicular weight with the co-exposure to PAEs and MPs.	↑ SOD ↑ MDA	[24]
Female Wistar rats	PS	0.5 μm	Beads	90 d	Presence of PS particles in GCs. Increased apoptosis of GCs. Increased fibrosis in ovary mediated by Wnt/β-Catenin signalling pathway. Alteration of follicle morphology and quantity. Reduced AMH levels.	↑ ROS and ↑ MDA ↓ SOD, ↓ CAT and ↓ GPx	[23]
	PS	0.5 μm	Beads	90 d	Presence of PS particles in GCs. Fewer growing follicles. Reduced AMH level. Pyroptosis and apoptosis of GCs mediated by NLRP3/Caspase-1 signalling pathway.	↑ MDA ↓ CAT, ↓ SOD and ↓ GPx	[96]
Male Wistar rats	PS	0.5 µm	Beads	90 d	Disruption of blood-testis barrier. Histological alteration in testis. Apoptosis of spermatogenic cells. Decreased sperm motility, concentration and quality.	↑ MDA ↓ CAT, ↓ SOD and ↓ GPx	[151]

8-OHdG: 8-hydroxydeoxyguanosine; ACP: acid phosphatase; AMH: anti-Müllerian hormone; CAT: catalase; d: days; GCs: granulosa cells; GPx: glutathione peroxidase; GR: glutathione reductase; GSH: glutathione; GST: glutathione S-transferase; h: hours; LDH: lactate dehydrogenase; MDA: malondialdehyde; MPs: microplastics; NPs: nanoplastics; PA: polyamide; PAEs: phthalates esters; PE: polyethylene; POPs: persistent organic pollutants; PP: polypropylene; PS: polystyrene; PVC: polyvinyl chloride; ROS: reactive oxygen species; SDH: succinate dehydrogenase; SOD: superoxide dismutase; TBT: tributyltin.

## 5. Conclusions and Recommendations for Future Directions of Research on Reproductive Alterations by Micro/Nanoplastics

According to the studies reviewed, OS is the leitmotif underlying the toxic effects determined by MPs/NPs on reproduction. The alteration of the antioxidant defence mechanisms and the increase in ROS production causing the OS may trigger gene instability, impair immune response, and induce reproductive abnormalities.

Concerns arise relative to the experimental conditions adopted and the achieved results. We noticed a mismatch between the concentrations, type, and size of MPs/NPs used in experimental conditions and those detected in environmental abiotic and biotic matrices. In aquatic models, the MPs/NPs concentrations employed in the water are much higher than those found in the environment, in freshwater, and even in heavily populated and industrialized coastal areas. At low concentrations/doses, MPs/NPs have negligible toxic effects and may even mitigate the negative impact of other contaminants in the case of co-exposure [156]. Following the hormesis principle, a biphasic concentration/dose–response relationship may be observed. Organisms’ exposure to low and constant concentrations (below the toxicological threshold) of a stressor may induce adaptive mechanisms. Thus, over time, if the detrimental conditions are sustained, a positive outcome is observed in the physiological function. A high enough stressor concentration (above the toxicological threshold) may instead result in negative effects [156,157], though an adaptive response has been observed in some species (i.e., freshwater gastropods).

In aquatic ecosystem, MPs/NPs mainly consist of fibres, followed by fragments and, to a smaller extent, by other shapes such as beads, films, and foams. However, most studies focused on spherical particles and on PS as the most frequent polymer type.

The literature data suggested that MP/NP-induced toxicity may increase when experimental procedures are based on small dimension particles with a positive charge, in addition to high concentrations. Further, the prevailing particle size used in reproductive studies is in the range 1–100 µm, and hence smaller than those frequently detected in the environment (see [158,159] and references therein). The majority of examined studies report the effects of virgin MPs/NPs manufactured by chemical companies and rarely those of weathered plastics.

Many studies are carried out on marine species and rarely on mammals, terrestrial, and/or wild species. The use of wild species for the evaluation of MPs/NPs toxicity in reproductive systems may nevertheless present several challenges. Wild organisms are exposed to different types of external stressors in the environment, making it extremely difficult to address the reported alterations to a specific stressor. Moreover, other factors can deeply impact the stressor’s toxicity: age, sex, background history, and environment.

Besides PS and the other few polymers usually examined, further studies are needed to investigate other MP/NP types, testing mixtures of MPs/NPs in the appropriate proportions, mirroring natural environmental conditions. It is worth noting that PE is the most common polymer detected in environmental samples. Pristine and aged plastics must be considered, adopting long-term exposure conditions, to better evaluate the chronic toxic effects that reflect the continued exposure of living beings.

All these discrepancies (between experimental and natural conditions) make it difficult to assess the toxic effects of MPs/NPs, and they do not allow to draw any firm conclusions about the reproduction toxicity of MPs/NPs. It has been evident that exposure to MPs/NPs can trigger toxicity pathways, among which inflammation and OS play a pivotal role; OS-related signalling pathways involved in immune and endocrine alterations have been observed. Indeed, ROS overproduction, MDA generation, altered expression of antioxidant enzymes genes, and OS-related genes have been reported in both aquatic and terrestrial species, and are related to reproductive damage. This was frequently determined by impaired spermatogenesis and reduced sperm quality, altered ovarian follicles morphology and quantity, and reduced reproductive parameters, among which are the survival and development of offspring. Moreover, reduced levels of hormonal and enzymatic regulators (i.e., testosterone, AMH, LDH, and SDH) have been observed, along with histological alterations in male and female gonads.

Very few studies have investigated the role of antioxidant substances on MP/NP-induced toxicity and reproductive impairment. They focused on NAC, evidencing its capability to counteract oxidative damage through a reduction in ROS production and, in turn, the mitigation of several effects related to OS.

Notably, the lack of (i) adequate standard sampling protocols, (ii) identification of appropriate species for MPs/NPs monitoring, (iii) development of standardized analytical methods for their detection in tissues, including those of the reproductive system, and (iv) uniformity in reporting units for both monitoring and toxicity studies, must be filled. The harmonized laboratory procedures for MPs/NPs detection would allow the comparison of available data on the occurrence of plastics in living organisms and the environment, as well as the risk assessment for human health. The presence of MPs in foetal and maternal placenta and in chorioamniotic membranes provides new light on the issue concerning human exposure to MPs. It is a matter of great concern, based on the crucial role of the placenta in supporting foetal development and, at the same time, providing an interface between the internal and the external environment. However, if the presence of MPs in human placenta is harmful to pregnancy, it must be investigated to determine the involvement of the immune system and inflammation. In fact, the results collected to date do not allow any extrapolation to mammal and human health but only lead us to hypothesize about the negative effects on male and female reproductive functions, which need to be elucidated.
